# The significance of exosomes in the development and treatment of hepatocellular carcinoma

**DOI:** 10.1186/s12943-019-1085-0

**Published:** 2020-01-04

**Authors:** Xin Li, Chuanyun Li, Liping Zhang, Min Wu, Ke Cao, Feifei Jiang, Dexi Chen, Ning Li, Weihua Li

**Affiliations:** 1 Beijing Youan Hospital, Capital Medical University, Beijing, China; 2 Beijing Institute of Hepatology, Beijing Youan Hospital, Capital Medical University, 8 Xitoutiao, Youanmenwai,Fengtai District, Beijing, 100069 China; 3 Department of Maternity, Yanan University Affiliated Hospital, Yanan, China

## Abstract

Hepatocellular carcinoma (HCC) is the most commonmalignancy. Exsome plays a significant role in the elucidation of signal transduction pathways between hepatoma cells, angiogenesis and early diagnosis of HCC. Exosomes are small vesicular structures that mediate interaction between different types of cells, and contain a variety of components (including DNA, RNA, and proteins). Numerous studies have shown that these substances in exosomes are involved in growth, metastasis and angiogenesis in liver cancer, and then inhibited the growth of liver cancer by blocking the signaling pathway of liver cancer cells. In addition, the exosomal substances could also be used as markers for screening early liver cancer. In this review, we summarized to reveal the significance of exosomes in the occurrence, development, diagnosis and treatment of HCC, which in turn might help us to further elucidate the mechanism of exosomes in HCC, and promote the use of exosomes in the clinical diagnosis and treatment of HCC.

## Key points

- Liver is a multicellular organ that requires intercellular communication for implementing its function.
- The function of exosomes in intercellular communication has been acknowledged.
- During the formation of HCC, the tumor cells communicate with all kinds of liver cells to promote growth of HCC and metastasis, exerting a huge effect on exosomes.
- Because of the differences in HCC stage, different biomarkers are found in exosomes. Also exosomes can be used as carriers to release several substances. Some of these can regulate signal transduction pathways between HCC cells, and others can be used as drugs because of external membrane protection.

## Background

Hepatocellular carcinoma (HCC) is the third most common malignancy in the world, accounting for 85–90% of primary liver cancer [1–3]. The annual incidence of new cases with HCC is estimated to be about 841,000 [4]. Hepatitis B virus (HBV) infection is considered as the main risk factor for HCC development in China [5]. Despite new breakthroughs in imaging technology, chemotherapy, interventional radiology, surgical techniques, and liver transplantation in the recent years, the prognostic rate of patients with advanced liver cancer still remained poor, and there is no effective treatment method till date for it [6–8]. Currently, the 5-year survival rate of HCC is not more than 20% [9, 10] and early diagnosis can significantly reduce the mortality in patients with HCC [11]. Therefore, the major methods to improve the total survival rate of HCC include improvementof the early diagnostic rate and explore the detailed formation mechanisms of HCC.

Exosomes are small nano-sized vesicles that transport biologically active molecules between cells, regulate microenvironment and immune system between cells by a variety of biomolecules (such as proteins, RNA and DNA) [4, 12, 13], and the genetic and epigenetic mechanisms of cells [14]. Studies have shown that exosomes not only initiates the downstream signals to target cells, but also transfers the genetic material to downstream cells, providing intercellular communication with a new mechanism [4]. It has been reported that different types of exosomal compositions provided by databases such as Vesiclepedia, EVpedia, and Exocarta were found in different cells with similar physiological or pathological conditions [15–17]. In addition, exosomes are also involved in angiogenesis, metastasis of tumor cells and transformation of normal cells into tumor cells [18]. Many types of cells such as mesenchymal cells, immune cells, and tumor cells may induce the release of exosomes, and increase in these cells indicate that exosomes are involved in tumorigenesis, development, metastasis, immune escape, and drug resistance [19]. Moreover, tumor-derived exosomes contain a large number of cancer-related serological markers, such as miRNAs, which could be used for detecting early HCC [20]. Despite several breakthroughs in the field of exosomes, the concrete biological role of exosomes has not been fully figured out. In this review, the source, structure, isolation of exosomes, and their impact and clinical application in HCC were described.

## The source and function of exosomes and the method for isolation of exosomes

**Fig. 1:**
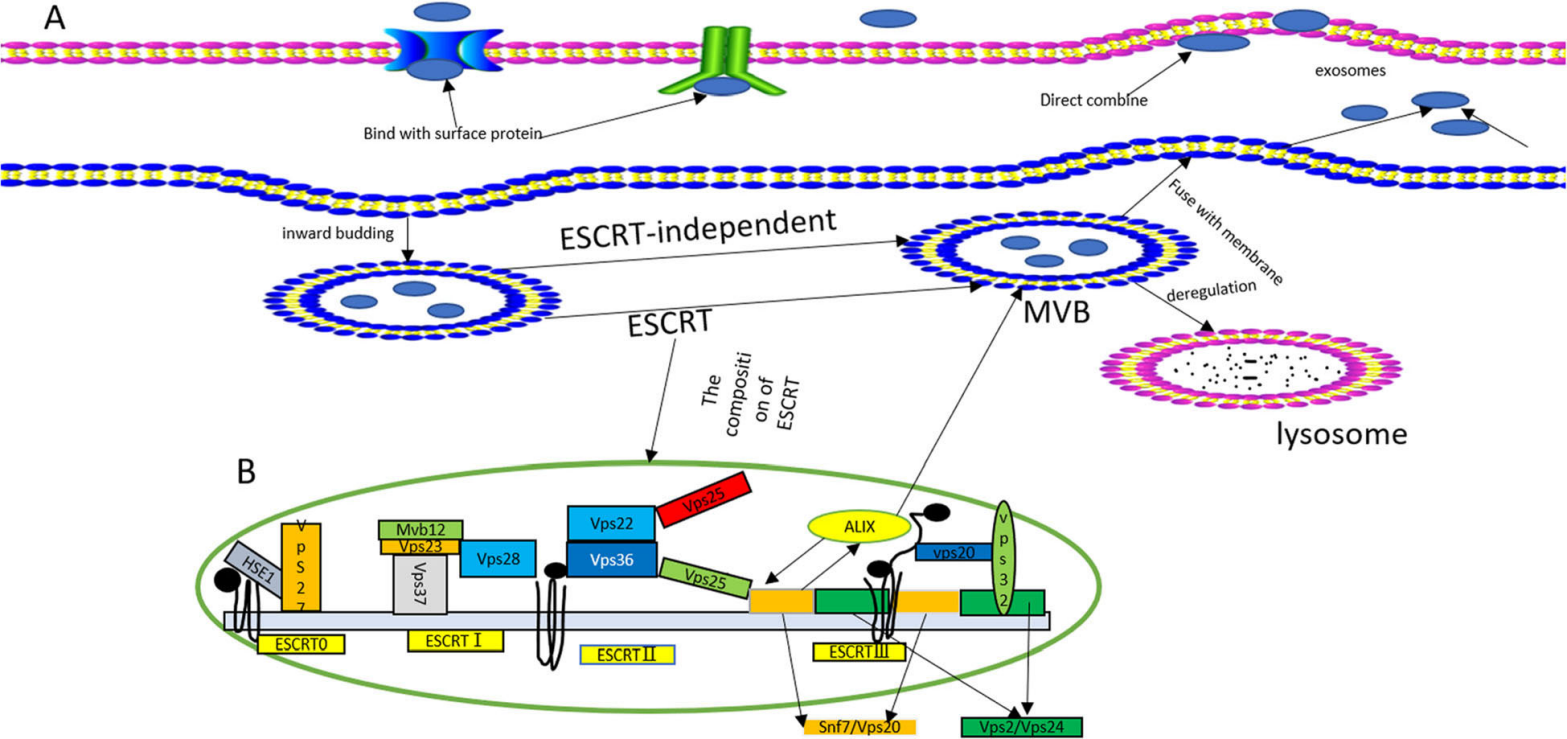
**a** Biosynthesis of exosomes. Firstly, the inward budding of cell membrane forms early endosomes. Secondly, the multivesicular bodies (MVBs) are generated by further inward budding of endosomes and several miRNAs, proteins and other selected substances are incorporated. Finally, the MVBs either fuse with cell membrane, leading to inclusion of extracellular DNA, or fuse with lysosome, causing degradation of biological information containers of MVBs. The mechanism of the formation of exosomes is depicted in detail. **b** The concrete structure of the 4 kinds of ESCRT (ESCRT 0, I, II, and III0), and ALIX, a cytosolic protein, interacts with ESCRT-III subunit SNF7, finally combining with MVBs [33]

Exosomes were first discovered by Johnstone RM et al. in 1983 in mature sheep reticulocytes and named them as exosomes. They were initially perceived as cellular “debris” [21]. In 1996, Raposo et al. have confirmed the significant role of exosomes in antigen presentation from B cells, causing T cells responses [22]. So, the exosomes have gradually drawn the attention of several researchers, and is now considered to play an important role in the diagnosis and therapy of tumors. Exosomes are small phospholipid bilayer membrane nanovesicles that are formed by segregation of intracellular poly vesicles with cell membranes, followed by releasing into the extracellular space [23]. The detailed formation of exosomes is presented as follows. To begin with, the inward budding of cell membrane confirms the early endosomal stage [24], followed by the generation of multivesicular bodies (MVBs) by further inward budding of early endosomes and several miRNAs, proteins and other selected substances [25]. Finally, the MVBs either fuse with cell membrane, leading to the inclusion of extracellular DNA [26, 27], or fuse with lysosome, inducing the degradation of biological information containers in MVBs [28]. The endosomal sorting complex required for transport (ESCRT) mainly guides special molecules into the exosomes of MVBs, and is regarded as an important mechanism of synthesis [29, 30]. The ESCRT mainly contains4 core ingredients (ESCRT 0, I, II, and III), wherein the primary function is to provide ubiquitinated proteins to induce lysosomal degradation and protein reusing [31]. In the above process, ALG2-interacting protein X (ALIX), an accessory protein, plays an important role in interacting with ESCRT-III subunit SNF7, and then combines with MVB [32]. There are other mechanisms discovered by the researchers and are considered as ESCRT-independent, as they play a big role in this procedure [24]. Due to less understanding onESCRT-independent mechanisms, a great effort should be made to outline the detailed role of ESCRT-independent mechanism. The concrete mechanism of the formation of exosomes is clearly presented in Fig. 1.

Exosomes can be synthesized by any cells, such as B lymphocytes, T cells, mast cells, dendritic cells (DC), etc. and might be secreted to enter into other cells to carry out their function [34, 35]. Exosomes contain biologically active substances including proteins, RNA, DNA, cholesterol, ngosine, and so on, which range in size from 50 to 140 nm [36–38]. The density of exosomes is about 1.13~1.19 g/ml [39]. Exosomes are abundant in human body, and can be found in biological fluids including urine, tears, plasma, breast milk, and cell culture supernatants [40–42]. Emerging evidence indicates that tumors during growth processing can secret exosomes. For example, Yang L illustrated a new function of long noncoding ribonucleic acid (lncRNA HOTAIR) that induces MVBs for transporting them to plasma membrane, further propelling the release of exosomes from HCC [43].

The isolation of exosomes mainly involves5 methods, which include differential ultracentrifugation [44, 45], polyethylene glycol (PEG) precipitation [46, 47], sucrose and iodixanol density ultracentrifugation [38], immunoaffinity (IAC) capture [48], and size exclusion chromatography [49, 50]. In this review, we mainly introduced differential ultracentrifugation, as it is considered as gold standard for isolating exosomes [51]. Firstly, centrifugation at 300 g for 10 min is used to remove cellular debris. Secondly, the microvesicles were removed by centrifugation at 10,000 g for 30 min. Thirdly, ultracentrifugation is performed at 1,00,000 g, for 90 min at 4 °C. Fourthly, the supernatant is discarded, followed by the addition of exosomal pellet to 1× PBS, and then washing it by ultracentrifugation at 1, 00,000 g for 90 min at 4 °C. Finally, the exosomes are resuspended in 500 μl 1 × PBS and stored at - 80 °C for further use [52]. But all these methods have their own limitations, and so further research of a more efficient method for isolating exosomes is needed.

## The role of exosomes in the process of chronic hepatitis B to hepatocellular carcinoma

**Fig. 2:**
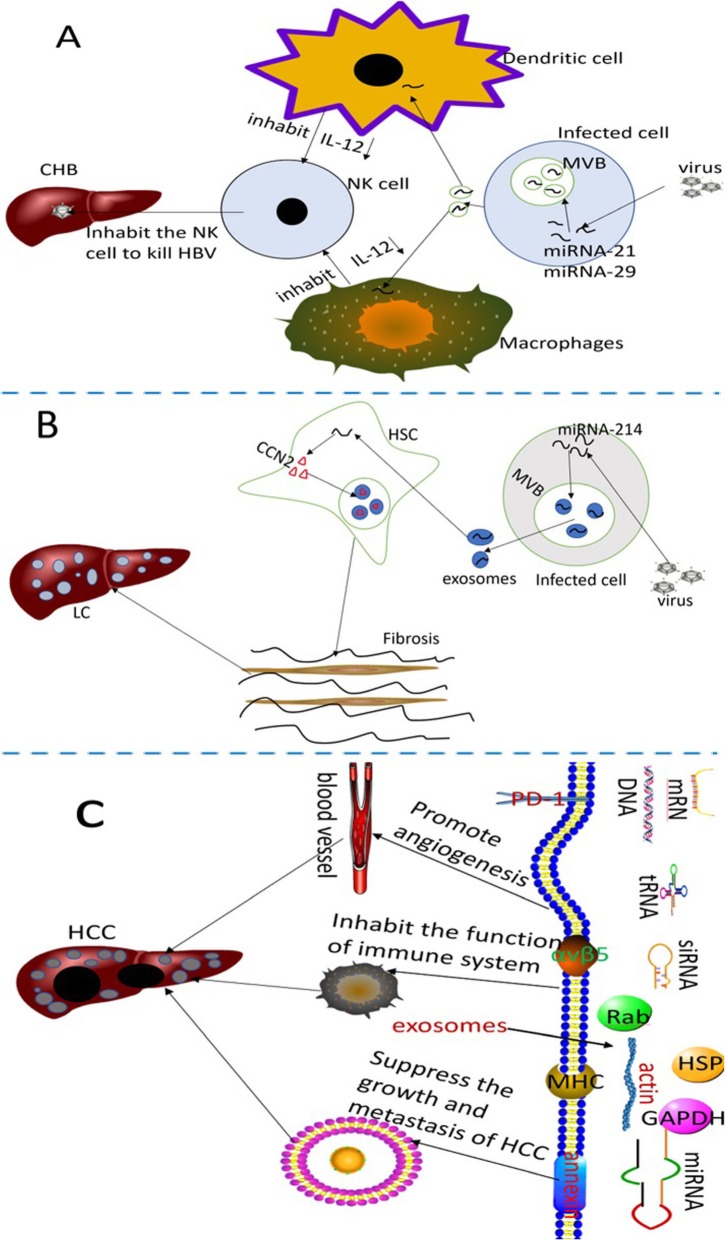
The detailed mechanism of the role exosomes played in the process of CHB to HCC. **a** After infection with HBV, the viruses using exosomes secrete some substances that lead to chronic infection with HBV. **b** During the chronic infection with HBV, the viruses stimulate all kinds of cells to release exosomes, leading to liver cirrhosis (LC). **c** The concrete mechanism of exosomes during the formation of HCC

Several normal liver cells (such as hepatocytes,stellate cells and immune cells) can secrete exosomes, and meanwhile, these extracellular vesicles mediate normal communication between liver cells, maintaining liver homeostasis [53]. According to Liu WH et al., the amount of serous exosomes in the cirrhotic stage, early HCC stage and late HCC stage is significantly higher than normal liver,liver degeneration and liver fibrosis, indicating that exosomes have high capability in the early detection of HCC [54]. Emerging evidence indicates that exosomes participate in the spread, immune regulation and antiviral response when the cells are infected by the viruses [55]. In china, the main reason for the cause of HCC is CHB, and recent study by Kouwaki T et al. demonstrated that the infected virus in HCC induces exosomal miRNA-21 and miRNA-29, inhibiting macrophages and dendritic cells and releasing IL-12. It is widely accepted that IL-12 activates natural killer (NK) cells, thereby undermining the innate immune responses [56]. This may in turn cause chronic infection of hepatitis B (Fig. 2a), and long history of CHB may bring the effect of Hepatic fibrosis. It is evident that the hepatic stellate cells (HSCs) transdifferentiates into myofibroblasts in response to certain stimuli, and this is associated with the pathogenesis of hepatic fibrosis and the development of HCC [57]. According to the research proposed by Angeles Duran et al.,p62/SQSTM1, a negative regulator of liver inflammation and fibrosis, promotes Vitamin D receptor (VDR) signaling activation in HSCs, and mediates retinoid X receptor,which in turn promotes heterodimerization during the inhibition of liver inflammation and fibrosis [58]. The above processes make a great difference in recruiting the target gene. Also, loss of p62 expression in HSCs increases myofibroblastic differentiation, while suppresses fibrosis and inflammation via VDR agonists in chemically-induced murine fibrosis and tumor models. Carcinoma-associated fibroblasts (CAFs) are most frequently observed in human carcinomas, and involve alpha-smooth muscle actin-positive myofibroblasts and actin-negative fibroblasts that promote the growth and progression of tumor [59]. Although the critical role of p62/SQSTM1 and CAFs in liver inflammation, fibrosis and tumor progressionhas been discovered previously, research on p62/SQSTM and CAFs in exosomes is still lacking. It is widely accepted that exosomes are used as carriers to block several signal transduction pathways [60], and hence much attention has been paid on the relationship between p62/SQSTM, CAFs and exosomes in order to explore whether they could prevent liver inflammation, fibrosis and tumor progressionthrough blocking signal transduction pathways. Chen L et al. have discovered that the exosomes can utilize miR-214 to interplay cellular shuttling in order to regulate the formation of connective tissue growth factor 2 (CCN2) [61]. Overexpression of CCN2 by exosomes in HSCs is found in the activation process of liver fibrosis [62, 63] (Fig. 2b). After several years, hepatic fibrosis proliferates into HCC. Emergingevidence proves that exosomes play a bigger role in this stage. The lipid components of exosomes not only participate in a variety of HCC biological processes, but also protect tumor-derived exosomes from enzymatic degradation [64]. The abundant protein Rab GTPase, annexin and similar exosomes could be used for HCC membrane transportation and fusion [65, 66]. Heat shock proteins such as HSP70, HSP60 and HSP90 could be used for early diagnosis and treatment of liver cancer [67]. Exosomal proteomics indicate that exosomal integrin αvβ5 showed close association with liver metastasis [68] (Fig. 2c). Tumor susceptibility gene 101 protein (TSG101), major histocompatibility complex (MHC) molecules and the ESCRT-III binding protein ALIX might be used as biomarkers for diagnosis [69, 70]. In addition to this, many nucleic acids, including messenger RNA (mRNA), microRNAs (miRNAs), long noncoding ribonucleic acid (LncRNA) and DNA, regulate cell genetics as well as epigenetics [71]. The process of CHB to HCC is displayed vividly in Fig. 2.

## The relationship between exosomes and hepatocellular carcinoma

Emerging evidence indicates that exosomes play a significant role in the formation and metastasis of HCC and the substances in exosomes are also used for clinical diagnosis and treatment. There are many types of liver cells, and so the intercellular communication is indispensable for liver cells to regulate and associate with each other [72]. Exosomes are novel research hot spots that play a critical role in intercellular communication [73]. In the next part, a thorough introduction on how the exosomes exert their role in HCC and how clinical exosomes are used as diagnostic and therapeutic tools is discussed.

### Exosomes regulate the growth and metastasis of HCC

Increasing evidence reveals that exosomes could participate in the growth and metastasis of HCC, such as by secreting miRNAs or other substances by regulating the growth and metastasis of HCC. The major reason for difficult treatment and poor prognosis is due to intrahepatic and distal metastasis [4], and so it is imperative to make clear the detailed mechanism of HCC. HCC cells secrete miRNA-21, resulting in the activation of PDK1/AKT signaling in HSCs. This in turn transforms HSC cells into CAFs. Activated CAFs secrete cytokines such as vascular endothelial growth factor (VEGF), matrix metalloprotein 2(MMP2), MMP9, bFGF and TGF-β to further promote cancer development [74]. In addition, exosomes containing miRNA-21 and miR-29a bind to toll-like receptor (TLR) in immune cells, activate NF-κB pathway in TLR, secrete a series of inflammatory factors, and promote tumor growth and transfer [75]. Jinxing W et al. found that HCC-derived exosomes promoted growth, metastasis and invasion of tumor cells in a co-culture experiment. In transporting the miRNAs to the recipient cell, Vps4A acts as an important negative regulator of exosomes. Through small RNA sequencing, Vps4A could regulate growth and metastasis of hepatoma cells by controlling the secretion and uptake of miRNAs [76]. Fang T et al. found that highly metastatic HCC cells produce exosomes containing miR-1247-3p, causing activation of β1-integrin-NF-κB signaling pathway by CAFs. CAF further promotes the development of cancer by activating IL-6 and IL-8. It can be seen that elevated exosomes of serum miR-1247-3p could promote lung metastasis in HCC patients [77]. Li B and other researchers have first discovered in an experiment that lncRNA FAL1 was upregulated in HCC exosomes, and meanwhile it could transfer to other HCC cells for promoting growth and migration of HCC [78]. Except HCC cells, other cells also can secret exosomes to promote HCC growth and reduce DNA damage. For example, Zhang H et al. in his research demonstrated that adipocytes could release exosomal circular RNAs (circRNAs) by reducing miR-34a and activating USP7/Cyclin A2 signaling pathway in tumor growth and reduce DNA injury [79].

### The role of exosomes in microcirculation of liver cancer

#### Effect of exosomes on hepatocellular carcinoma angiogenesis

Tumor growth requires blood vessels to provide a variety of nutrients. HCC is a highly angiogenic cancer, and VEGF plays a great role during the disease process. A recent study showed that mesenchymal stem cell (MSC)-derived exosomes serve as an important mediator of intercellular communication and inhibit tumor angiogenesis by down-regulating VEGF [80]. Xue Jia Lin et al. found that miR-210 present in exosomes, which is secreted by HCC cells, could be transferred to endothelial cells to promote tumor angiogenesis by targeting SMAD4 and STAT6 [81]. Hiroshi Yukawa et al. found that HepG2-exosomes express NKG2D(an activated receptor of immune cells), and HSP70 (a stress-related heat shock protein). Both these act on VEGF receptors, leading to angiogenesis [82]. Fang JH et al. found that miR-103 in exosomes secreted by HCC increase vascular permeability and promote liver cancer metastasis by acting on endothelial cells [83]. Lee HYand other researchers have found that the expression of EIF3C could enhance HCC cells to secrete exosomes, causing tube formation of HUVEC cells and tumor growth, eventually leading to tumor angiogenesis [84]. Although HCC is a kind of high-vascular solid tumor, hypoxia also plays an important role in the formation of this cancer [85]. Thus, the two major problems that need to be solved include inhibition of angiogenesis and alleviation of hypoxia. Research regarding the role of exosomes under hypoxic conditions, and also the mechanism about angiogenesis is limited. Therefore, more efforts should be made on these two aspects for promoting further research on HCC.

#### Exosomes participate in liver cancer epithelial-mesenchymal transition (EMT)

Epithelial-mesenchymal transition (EMT) refers to the loss of polarity of epithelial cells and disruption of connections between cells, thereby transforming into cells with an interstitial phenotype [86]. EMT is classified into two types, complete EMT and partial EMT. Complete EMT is observed in individuals with vital role of it in metastasis initiation. Karaosmanoğlu Oand their group demonstrated reduction of E-cadherin and upregulation of ZEB2 in complete EMT, and meanwhile partial EMT involves increase of E-cadherin and decrease of vimentin and ZEB2 [87]. It is worth notifying that the EMT might causean increasein the cancer stem-like cells (CSCs), which refers to the highly tumorigenic subpopulation of tumor cells that exists at the top of the hierarchical tumor cell society [88, 89], leading to tumor heterogeneity and therapeutic resistance [89]. Emerging evidence showes that EMT can lead to tumor migration and metastasis [90]. Chen L et al. found that MHCC97H-derived exosomes could initiate EMT of HLE and Hep3B cells via MAPK/ERK signaling pathway. By down-regulating Rab27a, excretion of MHCC97H-derived exosomes could be reduced, and EMT of parental MHCC97H cells could also be promoted [91]. Another research by Wang C et al. indicated that Wnt/β-catenin signaling pathway miRNA25 was initially activated, and miRNA then directly inhibits Rho GDP dissociation inhibitor alpha (RhoGDI1). Reduction of RhoGDI1 could lead to upward expression of snail, eventually causing EMT [92]. In relation to this, Tang et al. have put forwarded an in vitro experiment that promotes cell proliferation, migration, and invasion in vitro by CTNND1(delta-catenin), and promotes HCC cell tumor formation and metastasis by CTNND1in vivo. According to a more thorough assay, CTNND1 indirectly enhanced Wnt/β-catenin signaling to promote HCC metastasis. They also discovered that the expression of CTNND1showed a strong association when compared with β-catenin, WNT11, Cyclin D1, and BMP7 expressions in human HCC specimens. Knockdown of CTNND1 expression led to mesenchymal-epithelial transition (MET),but overexpression of it caused EMT and increased the potential of HCC metastasis [93, 94]. Studies on this topic are limited, but many evidences have proved that the functional molecules carried by tumor-associated exosomespromoted mesenchymal-associated gene expression and further induced EMT [95, 96]. So, it is necessary to perform a deeper exploration in the process of EMT.

### The role of exosomes in the immune regulation of liver cancer

In recent years, the use of immune cells that target tumors has become a research hotspot. In a recent study conducted by Lu Z et al., AFP-rich exosomes elicited a specific anti-tumor immune response, providing a new vaccine-free approach for treating HCC [97]. Rao Q also reported that exosomes derived from HCC antigens could elicit a stronger immune response than cell lysates. When using mouse cell-derived exosomes for HCC treatment, the tumor immune microenvironment showed significant improvement, such as increased levels of T cells and γ-IFN, decreased levels of IL-10 and TGF-β. We concluded that tumor cell-derived exosomes (TEX) that are expressed by HCC antigens might trigger a strong immune response in DC, thereby improving the immune microenvironment of HCC [98]. Liu J et al. found that endoplasmic reticulum stress could trigger the release of exosomes, and upregulate the expression of PD-1 molecules in macrophages in liver cancer cells. The exosome miR-23a-PTEN-AKT pathway was activated, which then inhibited the function of T cells [99]. It is widely accepted that macrophages play a significant role in innate immune response, and mainly the classical (M1) macrophages lead to anti-tumor activity. But alternate (M2) macrophages mainly promote tumorigenesis and tumor growth [100, 101]. A comparison experiment by Xue Liet al. demonstrated that knockdown of lncRNA UC339 in THP-1 cells led to M1 increase, and overexpression of lncRNA TUC339 in THP-1 cells caused M2 increase [102].

## Use of exosomes in the diagnosis and prognosis of liver cancer

Due to spatial heterogeneity and temporal heterogeneity of tumor, traditional method of tissue specimen biopsy cannot obtain the full and dynamic information of tumor tissues. In recent years, a novel diagnostic technology named “liquid biopsy” has been emerged to overcome the shortcomings of traditional tissue biopsy [103, 104]. In the protection of phospholipid bilayer membrane, the exosomal substances cannot be degraded by any enzyme, and so exosomes are considered as an appropriate diagnostic tool.

As the exosomes have many unique substances that can be expressed by tumor cells, we utilized them for the early detection of tumor. miRNAs are a class of conserved RNAs in the evolutionary history of humans and also participate in the development of liver cancer. According to the latest research, miRNA is considered as a potentially new and ideal marker [105]. Won Sohn et al. found that the levels of miR-18a, miR-221, miR-222 and miR-224 in exosomes of patients with HCC were lower than those in patients with HBV, indicating that these 3 miRNAs could be used as novel serum markers for detecting HCC [106]. Apart from miRNAs, lncRNAs are also used as biomarkers for clinical diagnosis of HCC [107]. Xiang Ma et al. discovered in an experiment that exosomes mediate the regulation of lncRNAs X-inactive-specific transcript in the expression of blood cells, and indicate that Xist expressed by mononuclear cells and granulocytes might act as valuable biomarkers in the diagnosis of female HCC patients [108].

Another study also reported that miR-30d, miR-140 and miR-29b showed significance in the survival of patients with liver cancer. Therefore, these exosomal miRNAs act as prognostic biomarkers for liver cancer and guide in the treatment of advanced liver cancer [109]. For proving the relationship of circulating exosomal noncoding RNAs (ncRNAs) in tumors, Lee YRet al. have conducted a series of experiments, which eventually showesa relationship of ncRNAs (miRNA-21 and lncRNA-ATB) with TNM staging and prognosis of HCC [110]. Xu Het al. indicates that serum exosomallncRNAs ENSG00000258332.1 and LINC00635 combined with serum AFP might be a promising method for diagnosis and prognosis of HCC [111]. Other experiment also supported that exosomal lncRNA acts as a prognostic factor in HCC, and lncRNA (LINC00161) significantly upregulates in HCC patients when compared to normal patients, which is well stabilized and specific [112]. In addition to lncRNA, circPTGR1, a circRNA, is particularly expressed in exosomes of 97 L and LM3 cells, and is increased in the serum exosomes of HCC patients. Wang G and his collaborator indicated that it could be used for clinical staging and prognosis [113].

## Application of exosomes in the treatment of liver cancer

HCC is not a sensitive disease to common chemotherapy. miR-122 could promote the sensitivity of HCC cells to chemotherapeutic drugs, and exosomes can be used as a biological carrier of miRNAs. These findings indicates that exosomes from self-body cells have less immunogenicity when compared with other vehicles [114]. Emerging evidenceshows the feasibility of applying exosomes as nanocarriers, for example, low immunogenicity, high biocompatibility, less toxicity and so on [115, 116]. At the same time, MSCs could secrete large amounts of exosomes. Researchers have found that transfection of miRNA-122 into adipose derived mesenchymal stem cells formed by MSCs could produce exocrine bodies containing miRNA-122, improving the miR-122-target gene expression and promoting sensitivity of cancer cells to chemotherapy [117]. Fang Wang and other researchers have found that stellate cell-derived EVs could load miR-335-5p. What makes us exhilarated is that miR-335-5 could be introduced into HCC cells to inhibit tumor growth and metastasis, which thereby provides a new treatment strategy for liver cancer [118]. Another research by Kenji Takahashiet al. have found that during the process of mediating chemotherapeutic stress response, RNAi-mediated knockdown of EVs (exosomes) lncRNAs could reduce the function and progression of tumor cells in HCC, which promotes the treatment of HCC [119].

## Conclusion and future prospects

Exosomes are involved in the occurrence, development, and metastasis of tumors, providing new clues for the treatment of HCC. We also found that many substances in exosomes including miRNAs serve as new biomarkers. It is also of great significance in improving the early diagnosis of HCC. Therefore, exosomes have become a hot research topic currently. This review has first introduced the role of exosomes in the development of CHB to HCC. Secondly, the mechanism of exosomes in tumor growth and metastasis is also discussed. The last but not the least, it is elucidated that exosomes could be used for clinical diagnosis and treatment. The function of the substance in HCC exosomes are conclued (Table 1). Although several studies have been put forwarded in investigating the relationship of exosomes and liver cancer, research on the formation mechanism of liver cancer by exosomes is still not deep enough. This is because of effective separation and specific detection of circulating exosomes in cancer cells [24, 125]. Meanwhile, the use of exosomes in studying the 4 serum markers of liver cancer (AFP, AFP-L3, GP73, and GPC3) has rarely been reported. Different researchers have drawn different views regarding the same exosomes. The major reason for this might be due to individual differences. So, environment, aging, gender, reason for HCC, and multi-center should be combined to produce more accurate results. For over the past several years, exosomes are used in immune therapeutic method only in 3 phase I clinical trials [126]. With more research conducted on exosomes, it is believed that exosomes could be successfully used in clinical diagnosis of early stage HCC in near future.

**Table 1:** The function of the substance in HCC exosomes

Components	Functions	First author/s	Year	References
Rab protein, GTPase, annexin	Membrane transport and fusion	CORDONNIER M	2017	[34]
miRNA				
miR-1247-3p	Promote lung migration of liver cancer	Fang T	2018	[77]
miRNA-210	Promotes angiogenesis	Lin XJ	2018	[81]
miR-103	Vascular permeability and metastasis	Fang JH	2018	[83]
miR-23a-3p	Inhibits the function of T-cell	Liu J	2019	[99]
miR-122	Improve the treatment effect	Lou G	2015	[117]
miR-335	Novel therapeutic strategy	Wang F	2018	[118]
microRNA-25-5p	Migration、Invasion	Liu H	2018	[122]
miR-320a-PBX3	Proliferation、metastasis	Zhang Z	2017	[123]
miR-718	Prediction the prognosis of HCC	Sugimachi K	2015	[124]
miR-665	Biomarker	Qu Z	2017	[125]
HSP				
HSP70, HSP60 and HSP90	Diagnosis and treatment	CORDONNIER M	2017	[34]
lncRNA HOTAIR	The release of exosomes	YANG L	2019	[45]
RNA and DNA	Regulation cell genetics and epigenetics	ZHANG X	2015	[71]
Vps4A	Tumor suppressor	Jin-xing Wei	2015	[76]
lncRNA				
lncRNA-ATB	Novel prognosis of biomarker and therapeutic targets	Lee YR	2019	[110]
LUCAT1 and CASC9	Biomarker	Gramantieri L	2018	[126]
circRNA				
circ-DB	Promote HCC growth and reduce DNA damage	Zhang H	2019	[79]
circPTGR1	Clinical stage and prognosis	Wang G	2019	[113]

*HCC* hepatocellular carcinoma, *miR/miRNA* micro ribonucleic acid, *HSP* heat shock proteins, *circRNA* Circular RNAs, *lncRNA* Long noncoding ribonucleic acid

## Data Availability

Not applicable

## Abbreviations

ALIX: ALG2-interacting protein X
CAFs: Carcinoma-associated fibroblasts
CCN2: Connective tissue growth factor 2
CHB: Chronic hepatitis B
circRNAs: circular RNAs
CSCs: Cancer stem-like cells
EMT: Epithelial-mesenchymal transition
ESCRT: Endosomal sorting complex required for transport
HCC: Hepatocellular carcinoma
HSCs: Hepatic stellate cells
HSP: Heat shock proteins
IAC: Immunoaffinity
LC: Liver cirrhosis
lncRNA: Long noncoding ribouncleic acid
MHC: Major histocompatibility complex
MMP: Matrix metalloprotein
MSC: Mesenchymal stem cell
MSCs: Mesenchymal cells
MVB: Multivesicular bodies
NK: Natural killer
PEG: Polyethylene glycol
Rho GDI: Rho GDP dissociation inhibitor alpha
TEX: Tumor cell-derived exosomes
TLR: Toll-like receptor
TSG101: Tumor susceptibility gene 101 protein
VEGF: Vascular endothelial growth factor
